# The Interplay between Metabolic Syndrome and Religious Fasting in Postmenopausal Women

**DOI:** 10.3390/nu15112478

**Published:** 2023-05-26

**Authors:** Anna Kokkinopoulou, Niki Katsiki, Ioannis Pagkalos, Nikolaos E. Rodopaios, Alexandra-Aikaterini Koulouri, Eleni Vasara, Sousana K. Papadopoulou, Petros Skepastianos, Emmanouil Dermitzakis, Maria Hassapidou, Anthony G. Kafatos

**Affiliations:** 1 Department of Preventive Medicine and Nutrition Unit, School of Medicine, University of Crete, 71003 Crete, Greece; kokkinopoulouan@gmail.com (A.K.);; 2 Department of Nutritional Sciences and Dietetics, International Hellenic University, 57400 Thessaloniki, Greece; 3 School of Medicine, European University Cyprus, Nicosia 2404, Cyprus; 4 Laboratory of Animal Physiology, Department of Zoology, School of Biology, Aristotle University of Thessaloniki, 54124 Thessaloniki, Greece; evasara@bio.auth.gr; 5 Department of Medical Laboratory Studies, International Hellenic University, 57400 Thessaloniki, Greece; 6 Department of Genetic Medicine and Development, Faculty of Medicine, University of Geneva, 1211 Geneva, Switzerland

**Keywords:** nutrient intake, blood lipids, postmenopausal women, metabolic syndrome, religious fasting, Christian Orthodox fasting

## Abstract

Religious fasting that involves abstinence from specific food(s) is part of many religions worldwide and has been gaining attention by the research community during the last years. The study aimed to investigate whether the periodic Christian Orthodox fasting mitigates the changes in body composition, dietary intake, and metabolic syndrome (MetS) in postmenopausal women. One hundred and thirty-four postmenopausal women aged 57.3 ± 6.7 years participated in this study. The Christian Orthodox fasting was followed by 68 postmenopausal women since their childhood, whereas 66 postmenopausal women were non-fasters. Data collection involved anthropometric, biochemical, clinical, and dietary information. Postmenopausal women who fasted according to Christian Orthodox Church recommendations had significantly higher mean fat free mass (45 vs. 44 kg, *p* = 0.002), hip circumference (104 vs. 99 cm, *p* = 0.001), and diastolic blood pressure (79 vs. 82 mmHg, *p* = 0.024). No other differences were found with regards to anthropometric data. Fasters also consumed significantly less fat (78 vs. 91 g, *p* = 0.006), as well as saturated (19 vs. 23 g, *p* = 0.015), monounsaturated (41 vs. 47 g, *p* = 0.018), and polyunsaturated fat (8.5 vs. 10 g, *p* = 0.023), trans fatty acids (0.5 vs. 2.3 g, *p* = 0.035), and cholesterol (132 vs. 176 g, *p* = 0.011). In terms of MetS features, non-fasters had more frequently elevated fasting blood glucose (11.8 vs. 24.2%, *p* = 0.039) and elevated blood pressure (13.2 vs. 36.4%, *p* = 0.041) compared with fasters. MetS was more common in non-fasters versus fasters with a marginal level of significance (30.3 vs. 23.5%, *p* = 0.052). Postmenopausal women who follow the Christian Orthodox fasting regime had lower fat intake, and no other difference in nutrient intake, compared with non-fasters. The latter were more likely to have MetS and some of its components. Overall, periodic abstinence from meat, dairy products, and eggs might play a protective role in postmenopausal women with regard to MetS.

## 1. Introduction

Central obesity plays a key role in inducing insulin resistance and developing metabolic syndrome (MetS) [1,2]. Changes in body structure, and more specifically increased fat accumulation and central obesity, have also been associated with menopause [3,4]. The menopause phase is also linked to different metabolic changes, including increased blood pressure (BP), dyslipidemia, and dysglycemia [5,6]. 

MetS is a cluster of metabolic disorders, namely central obesity, insulin resistance, hypertension, and dyslipidemia [7,8,9], and it is related to increased cardiovascular (CV) risk [10,11,12]. Healthy dietary intake alongside increased physical activity is recommended for MetS prevention and/or treatment [13,14]. According to a recent review by Santa and colleagues, a Mediterranean diet and a traditional Japanese diet, both characterized by low fat, sugar, and red meat intake, were negatively associated with MetS development [15]. This outcome was also supported by the results of the Longevity Blue Zone (LBZ) project revealing that populations from Ikaria, Greece [16,17] and Okinawa, Japan [18] have exceptional longevity and reduced incidents of chronic diseases.

Fasting has gained a lot of interest as a means of adopting a healthier lifestyle to prevent and treat cardiometabolic diseases, including MetS [19]. In addition, religious fasting has traditionally been followed by people of all faiths worldwide, but has also emerged as a healthy dietary pattern that can be adopted by individuals [20] with its effects being investigated during the past few years [21,22,23,24,25,26,27]. Christian Orthodox Church (COC) religious fasting, which is followed by the largest religious community worldwide [28], suggests abstinence from meat, dairy products, and eggs for 180–200 days per year, according to the fasting periods that are spread throughout the year [22]. During the fasting periods, consumption of seafoods and snails is allowed, together with the increased consumption of fruits, vegetables, legumes, and cereals. The COC fasting represents an interchange of plant-based to vegetarian diet and is considered as the underpinning of the Mediterranean diet [22,29]. 

The present study aimed to evaluate differences in anthropometry, dietary intake, and MetS presence between postmenopausal women who follow COC religious fasting and non-fasters.

## 2. Methods

### 2.1. Study Design and Population

This was a cross-sectional study conducted in Thessaloniki, the sub-capital of Greece. The design allowed for individuals to be recruited on a voluntary basis, after a call for volunteers through public universities and churches in the prefecture of Thessaloniki, Northern Greece. Ethical approval was provided by the Ethics Committee of the Alexander Technological Educational Institute of Thessaloniki. All participants gave their written informed consent and were free to withdraw from the study without any repercussions.

Four hundred and fifty-four individuals initially showed interest to participate in the study. Fifty-four did not qualify as fasters, and/or did not attend the scheduled appointment for all measurements. Thus, 400 individuals participated in the present study, equally divided to fasters and non-fasters (200 and 200, respectively).

Overall, there were 134 women, with a mean age of menopause beginning at 48.5 ± 3.7 years, that participated in the study. Among them, 68 women adhered to the COC fasting recommendations for a mean period of 34.0 ± 16.0 years, while 66 women were non-fasters. 

### 2.2. Outcome Measures

As part of the study protocol, anthropometric measurements, biochemical data, clinical history information, dietary intake data, and environmental information were collected based on the nutrition care process (NCP) approach [30,31]. All anthropometric measurements were performed by the same examiner, a trained nutritionist.

As far as the clinical history information is concerned, all participants answered an initial questionnaire with close-ended questions regarding medication and health issues. Body weight, height, waist, and hip circumference were measured, with participants in minimal clothing. The same calibrated equipment was used for all measurements; a TANITA digital scale (TANITA UM075, Amsterdam, The Netherlands), a TANITA stadiometer (TANITA HR001, Leicester, UK), a BODYSTAT bioimpedance analyzer (BODYSTAT 1500, Warwickshire, UK), and a SECA body girth tape (SECA 201, Hamburg, Germany). BP was also measured when participants were relaxed, with the use of a validated electronic BP monitor (Omron, Hoffman Estates, IL, USA).

The same nutritionist was also responsible for data collection on dietary intake via a detailed questionnaire that included three 24 h diet recalls, a monthly food frequency questionnaire (FFQ), and a validated questionnaire on eating habits and supplement use among other details. Finally, data on physical activity, lifestyle habits, and socioeconomic status were collected by a validated questionnaire used in the Greek population [32]. Venous blood was obtained for biochemical analysis, which included blood glucose and lipids. Biochemical data were analyzed in a certified lab. Appointments were scheduled via a phone call, during morning hours, with participants fasting after midnight on the day before the appointment. 

### 2.3. Metabolic Syndrome

MetS was defined by the joint criteria [4], requiring the presence of three or more of the following risk factors in women: waist circumference > 88 cm, BP ≥ 130/85 mmHg, high-density lipoprotein (HDL) cholesterol < 40 mg/dL, fasting blood glucose ≥ 100 mg/dL, and triglycerides (TG) ≥ 150 mg/dL [4].

### 2.4. Statistical Analysis

The SPSS statistical software v.21 (IBM, New York, NY, USA) was used for data analysis. Means, standard deviations (SD), and frequencies (%) were used to describe continuous and categorical data, respectively. Furthermore, *t*-test, ANOVA, and logistic regression analysis were performed. Statistical significance was set at a 2-sided *p*-value of 0.05.

## 3. Results 

Among 200 women that participated in the present study, 134 were postmenopausal with a mean age of 57.3 ± 6.7 years, mean weight 72.7 ± 11.1 kg, and mean body fat 38.7 ± 5.7%. With regards to education status, most of the menopausal women had secondary education (42.5%), followed by tertiary (35.8%). The majority of the participants were married (78.4%), while a small proportion was single (11.2%). Additionally, a high percentage of postmenopausal women did not smoke and/or quit smoking (79.1% and 0.7%, respectively), while 20.1% of them accounted as smokers. As far as other lifestyle habits are concerned, postmenopausal women tended to sleep for 6.6 ± 1.2 h per night and nap for 1.0 ± 0.7 h per day. A small percentage of them had any form of physical activity every day (27.6%), while the majority (41%) had moderate physical activity, accounting for 2–3 times per week. 

Among the 134 postmenopausal women, 68 followed the COC fasting regime since their childhood with a mean duration of fasting 34.0 ± 16.0 years, whereas 66 postmenopausal women did not, accounting for the non-fasters. Fasters had a mean age of 57.7 ± 7.1 years, mean menopausal age 49 years, mean weight 74.2 ± 11.1 kg, mean body mass index (BMI) 28.93 ± 4.12 kg/m^2^, and mean body fat 39.0 ± 5.7%. Non-fasters had a mean age of 56.9 ± 6.2 years, mean menopausal age 48 years, mean weight 71.1 ± 11.0 kg, mean BMI 27.56 ± 4.47 kg/m^2^, and mean body fat 38.4 ± 5.7%; no significant differences were observed between fasters and non-fasters in relation to these variables (Table 1). In contrast, fasters had significantly higher mean fat free mass (44.8 ± 3.9 vs. 43.2 ± 3.6 kg, *p* = 0.020) and hip circumference (103.9 ± 8.1 vs. 99.4 ± 7.5 cm, *p* = 0.001) compared with non-fasters. Diastolic BP (DBP) was significantly lower in fasters (79 ± 7 vs. 82 ± 8 mmHg, *p* = 0.024) with systolic BP (SBP) being non-significantly lower (fasters: 130 ± 12 mmHg, non-fasters: 131 ± 14 mmHg, *p* = 0.55) (Table 1).

Regarding socio-economic status, significant differences were found in family status (*p* < 0.001), with the majority of fasters (73.5%) being married or single (20.6%), while most of non-fasters were married (83.3%) or divorced and widowed (15.2%) (Table 1). Furthermore, significantly more non-fasters were smokers (37.9 vs. 2.9%, *p* < 0.001) compared with fasters, while the majority of fasters (95.6%) never smoked (Table 1). No differences between the two groups (i.e., fasters and non-fasters) were observed in BMI, way of eating and cooking, use of supplements/extra salt/diet products, and physical activity as shown in Table 1. Anthropometric data and lifestyle habits are summarized in Table 1. 

Analysis of the three 24 h dietary recalls revealed differences in terms of energy and macronutrient intake (Table 2). Energy, protein, and carbohydrate intake were similar between fasters and non-fasters (energy intake: 1453.73 ± 364.80 vs. 1580.86 ± 453.16 kcals respectively, *p* = 0.07; protein intake: 48.89 ± 17.74 vs. 54.70 ± 19.45 g, respectively, *p* = 0.07; carbohydrate intake: 150.94 ± 50.78 vs. 148.60 ± 53.90 g, *p* = 0.79). In contrast, fasters consumed significantly more monosaccharides (16.48 ± 12.65 g vs. 12.34 ± 8.28 g, *p* = 0.027), significantly less total fat (77.87 ± 25.76 g vs. 90.55 ± 26.94 g, *p* = 0.006), saturated fat (19.47 ± 8.49 g vs. 23.26 ± 9.24 g, *p* = 0.015), monounsaturated fat (40.99 ± 16.10 g vs. 47.18 ± 13.72 g, *p* = 0.018), polyunsaturated fat (8.56 ± 3.32 g vs. 10.36 ± 5.51 g, *p* = 0.023), trans fatty acids (0.57 ± 0.45 g vs. 1.20 ± 2.38 g, *p* = 0.035), cholesterol (131.77 ± 97.79 mg vs. 176.55 ± 104.28 mg, *p* = 0.011), and ω-6 fatty acids (4.83 ± 2.48 mg vs. 6.61 ± 4.36 mg, *p* = 0.004) compared with non-fasters.

Concerning biochemical analysis (Table 3), fasters had significantly lower mean blood glucose levels (86 ± 17 vs. 95 ± 25 mg/dL, *p* = 0.022) than non-fasters. Total cholesterol (*p* = 0.054) and TG (*p* = 0.057) were non-significantly lower in fasters (with a marginal level of significance).

When looking into the specific features of MetS (Table 4), significantly more non-fasters had elevated fasting blood glucose (8 fasters vs. 16 non-fasters, *p* = 0.039) and hypertension (9 fasters vs. 24 non-fasters, *p* = 0.041). Furthermore, fasters tended to have lower HDL cholesterol levels (32 fasters vs. 21 non-fasters, *p* = 0.61) and elevated waist circumference (49 fasters vs. 41 non-fasters, *p* = 0.55) but not elevated TG (22 fasters vs. 31 non-fasters, *p* = 0.58). Overall, non-fasters were more likely to have MetS than fasters (30.3 vs. 23.5%), but this difference was marginally significant (*p* = 0.052). 

Among fasters, 6 met zero components, 25 had one, 21 had two, 10 had three, 6 had four components, while no faster met all five components of MetS. The corresponding values for non-fasters were 8, 20, 18, 8, and 7, whereas 5 non-fasters had all five MetS features. 

Further analysis based on the presence or absence of MetS showed that postmenopausal women with MetS were older (59.5 ± 7 vs. 56 ± 6.5 years, *p* = 0.038), had lower education (49 vs. 29% up to secondary, *p* = 0.005), spent more hours in front of a screen (computer/phone) (2.5 ± 1 vs. 2 ± 0.5 h/day, *p* = 0.008), had lower physical activity during the week (64 vs. 31% never or less than 2 times per week, *p* < 0.001), and less free time workouts (23 vs. 43%, *p* = 0.031) compared with those without MetS. Furthermore, postmenopausal women with MetS had higher body weight (69.6 ± 9.3 vs. 81.1 ± 11.3 kg, *p* < 0.001), BMI (27 ± 3.7 vs. 31.5 ± 4.1 kg/m^2^, *p* < 0.001), body fat (37.1 ± 5.3 vs. 43 ± 4.5 kg, *p* < 0.001), fat mass (26.2 ± 6.7 vs. 35.3 ± 8 kg, *p* < 0.001), fat free mass (43.4 ± 3.6 vs. 45.7 ± 4 kg, *p* < 0.001), waist circumference (90.1 ± 11 vs. 102.3 ± 8.5 cm, *p* < 0.001), hip circumference (100.7 ± 7.6 vs. 104.3 ± 9 cm, *p* < 0.001), waist to hip ratio (0.9 ± 0.09 vs. 1 ± 0.08, *p* < 0.001), SBP (128 ±12 vs. 139 ± 12 mmHg, *p* > 0.001), DBP (78 ± 7 vs. 85 ± 8 mmHg, *p* < 0.001), total cholesterol (217 ± 45 vs. 235 ± 52 mg/dL, *p* = 0.048), TG (123 ± 72 vs. 240 ± 107 mg/dL, *p* < 0.001), and fasting glucose (85.3 ± 13.4 vs. 104 ± 31 mg/dL, *p* < 0.001), while having lower HDL cholesterol levels (61 ± 17 vs. 45 ± 16 mg/dL, *p* < 0.001) in relation to those without MetS.

ANOVA analysis found no significant differences between family status (*p* = 0.877), smoking habit (*p* = 0.235) and MetS prevalence. In contrast, lower education level (*p* = 0.014) and lower physical activity (i.e., those never being active or with less than two times weekly of physical activity) were significantly associated with MetS (*p* = 0.014 and 0.006, respectively). 

A logistic regression model that included age, family status, education level, smoking habit, BMI, and physical activity was used in both fasters and non-fasters to predict the probability of MetS in postmenopausal women. In fasters, age and BMI were significantly and positively related to MetS presence (*p* = 0.049 and <0.001, respectively). In non-fasters, only BMI was positively associated with MetS (*p* = 0.001). When logistic regression analysis was performed in all postmenopausal women, education, BMI, and physical activity were significantly linked to MetS presence (*p* = 0.020, <0.001, and 0.006, respectively). 

## 4. Discussion 

In the present cross-sectional study, postmenopausal women who followed the COC fasting consumed non-significantly less energy, protein, and carbohydrates, as well as significantly less total fat, saturated, monounsaturated, and polyunsaturated fat, trans fatty acids, cholesterol, and ω-6 fatty acids compared with non-fasters. Although low energy intake was noticed in both faster and non-faster menopausal women, high percentages of overweight and obesity are noticed in the two groups. This could be explained from the case of under-self-reporting dietary intake, which is a known drawback in epidemiological studies. Additionally, it could be explained from the results of the reported physical activity levels, which showed that 42.7% fasters and 51.5% non-fasters performed rarely or once per week any form of physical activity. These results should be further examined to investigate any associations between the nutrient content of the diet, the physical activity level, and the reasons for the low energy intake. Similar results were demonstrated by Bethancourt and colleagues, who showed that fasters decreased their energy, protein, and fat intake, as well as their saturated fat and trans fatty acids intake, during a COC 48-day fasting period [33]. Another study found that fasters had lower protein, fat, saturated fat, and trans fatty acids consumption during a fasting week compared with a non-fasting week [34]. Furthermore, fasters were reported to reduce their protein and fat intake, as well as saturated fats, trans fatty acids, and cholesterol during the three largest COC fasting periods [35,36]. Of note, according to a recent review by Silva and colleagues, low-energy diets could be recommended to postmenopausal women to prevent metabolic disorders, as well as a Mediterranean diet that could decrease their BP [37]. We believe that our study further explores these suggestions, since the COC fasting dietary pattern is part of the Mediterranean diet. 

Our study revealed that postmenopausal women who followed the COC fasting regime had non-significantly lower mean values of total cholesterol and TG compared with non-fasters. Similarly, total cholesterol was significantly decreased after a 43-day fasting period (187 vs. 173 mg/dL, *p* < 0.001) in a population (*n* = 36) in Egypt [38], as well as after a 48-day fasting period in US individuals (*n* = 99) [33]. Furthermore, a significant reduction in total cholesterol (346 vs. 80 mg/dL, *p* < 0.005) was reported in 36 Greek individuals who fasted for 40 days according to the COC fasting recommendations [39] and a 12.5% significant reduction (*p* < 0.001) in another population of 60 Greek individuals who were followed during the three biggest COC fasting periods [36]. TG were not statistically lowered (150 vs. 142 mg/dL, *p* = 0.10) in 49 Egyptian COC fasters who were followed during a 48-day period [40] and in 37 Greek COC fasters who were followed during a 40-day fasting period (*p* = 0.017) [41]. Similarly, in a study with 28 postmenopausal women, total cholesterol was decreased during a 3-month intervention period with a low-fat diet [42]. Additionally, increased consumption of wholegrain products and legumes, as components of a plant-based diet, were linked with improvements in cholesterol, glucose, and insulin [43]. 

Another finding of the present study is that postmenopausal women who followed the COC fasting recommendations had significantly lower mean fasting glucose levels compared with non-fasters. No other study reported significant reductions in glucose levels during fasting periods. For example, among 49 Egyptian fasters with type 2 diabetes, no change was observed in blood glucose values after the Easter fasting period that lasted 48 days [40]. Similarly, the 43-day Christmas period did not affect blood glucose levels in 36 Egyptian fasters [38], as well as the 1-week fasting period in 50 Greek fasters [44]. A reduction in fasting glucose was also noticed in 334 postmenopausal Mexican women in the Women’s Health Initiative Observational Study that showed higher levels of adherence to the DASH diet [45].

An interesting finding of the present study is that postmenopausal women who fasted had significantly lower mean DBP and non-significantly lower mean SBP compared with non-fasters. Menopause is associated with elevated BP [46]. Results from our study support that the COC fasting diet can decrease BP, thus proving a potential protective role of this dietary pattern. El-Sayed and colleagues found that, during all fasting periods, individuals who were fasters with hypertension statistically decreased their BP levels (139 vs. 130 mmHg, *p* < 0.001) when compared to non-fasters [40]. In accordance with our results, studies from Greek populations showed that after a 48-day fasting period (*n* = 38 fasters), SBP was significantly lowered (−0.5 mmHg, *p* < 0.001) [47]. Of note, vegetarian diets, which are based mainly on increased consumption of plant-origin foods, e.g., fruits and vegetables, are even more effective than lacto-ovo-vegetarian diets in reducing SBP (−2.66 mmHg, *p* < 0.001) and DBP (−1.69 mmHg, *p* < 0.001) when compared with omnivorous diets, as shown in a previous meta-analysis [48]. A study with 2208 American postmenopausal women showed that SBP was significantly lowered (−1.1 mmHg, *p* = 0.02) by a low-fat diet that was followed for 6 months [49].

In terms of MetS presence, we found that, in postmenopausal women, non-fasters were more likely to have MetS with a marginal *p* value (*p* = 0.052). Further larger studies with postmenopausal women will shed more light onto this finding. Such a benefit is of great importance, keeping also in mind that the COC fasting recommendations refer to 180–200 days per year. According to our knowledge, this is one of the first studies that focused on the effects of COC fasting on MetS and showed promising protective effects. In addition, studies that focus on vegetarian diets [50], such as the Adventist Health Study 2 [51] and the China Health and Nutrition Survey [52], have found that following a more plant-based dietary pattern may have a protective role against MetS, thus further supporting our study findings.

A limitation of the present study is the use of self-reported dietary intake (even if it was supervised by a nutritionist), which is a known limitation in the literature [53]. However, we used a combination of questionnaires in order to eliminate this issue. The sample size was small, and it can be considered as a convenience sample as it was not chosen through random selection, and thus future larger studies are needed to establish the observed associations. On the other hand, this is one of the first studies focusing on postmenopausal women who fasted according to the COC recommendations. Another strength is that the data were collected during a non-fasting period and thus represent the COC fasters’ health status throughout the year. 

## 5. Conclusions

It is becoming increasingly evident that COC fasting regime might have a protective role against cardiometabolic diseases, and potentially MetS. In the present study, among postmenopausal women, fasters consumed significantly less fat (total, saturated, monounsaturated, and polyunsaturated), trans fatty acids, and cholesterol compared with non-fasters. Fasters had also significantly lower fat free mass, fasting glucose levels, and DBP, but higher hip circumference. In terms of MetS components, non-fasters more frequently had elevated fasting blood glucose and elevated BP compared with fasters. MetS was more common in non-fasters vs. fasters with a marginal level of significance. These findings support a protective role of COC fasting in MetS prevention and treatment also in postmenopausal women. Future studies should be performed, with a larger sample size and/or with scheduled follow-up appointments, in order to investigate the long-term results of the COC fasting and reach definite conclusions.

## Figures and Tables

**Table 1:** Lifestyle habits and anthropometry of the two groups.

Variable	Fasters (*n* = 68)		Non-Fasters (*n* = 66)		*p*-Value
	Mean ± SD		Mean ± SD		
**Age (years)**	57.7 ± 7.1		56.9 ± 6.2		0.44
**Weight (kg)**	74.2 ± 11.1		71.1 ± 11		0.10
**Height (m)**	1.60 ± 0.05		1.60 ± 0.04		0.53
**BMI (kg/m^2^)**	28.9 ± 4.12		27.56 ± 4.47		0.06
**Body fat (%)**	39.0 ± 5.7		38.4 ± 5.7		0.55
**Body fat (kg)**	29.4 ± 8.2		27.8 ± 8		0.26
**Fat-free mass (kg)**	44.8 ± 3.9		43.2 ± 3.6		0.020
**Waist circumference (cm)**	95.3 ± 12.3		91.4 ± 10.8		0.056
**Hip circumference (cm)**	103.9 ± 8.1		99.4 ± 7.5		0.001
**WHR**	0.91 ± 0.1		0.9 ± 0.09		0.92
**SBP (mmHg)**	130 ± 12		131 ± 14		0.55
**DBP (mmHg)**	79 ± 7		82 ± 8		0.024
**Pulses (per min)**	69 ± 8		71 ± 9		0.10
	***n***	**%**	***n***	**%**	***p*-value**
**Education level**					0.80
Primary education	9	13.2	12	18.2	
Secondary education	31	45.6	26	39.4	
Tertiary education	24	35.3	24	36.4	
Master’s/Doctoral	4	5.9	4	6.1	
**Family status**					<0.001
Single	14	20.6	1	1.5	
Married/Living together	5	73.5	55	83.3	
Divorced	4	5.9	5	7.6	
Widowed	-	-	5	7.6	
**Smoking habit**					<0.001
Yes	2	2.9	25	37.9	
No—never	65	95.6	41	62.1	
No—quit smoking	1	1.5	-	-	
**BMI**					0.18
Underweight (<18.5 kg/m^2^)	-	-	-	-	
Normal weight (18.5–24.9 kg/m^2^)	11	16.2	23	34.8	
Overweight (25–29.9 kg/m^2^)	35	51.5	21	31.8	
Obesity (≥30 kg/m^2^)	22	32.4	22	33.3	
**Supplement use**					0.92
Yes	15	22.1	15	22.7	
No	53	77.9	51	77.3	
**Way of eating**					0.07
Anxious	2	2.9	2	3.0	
Slowly	27	39.7	37	56.1	
Quickly	27	39.7	20	30.3	
Regular	12	17.6	7	10.6	
**Way of eating—company**					0.29
Sitting alone	13	19.1	11	16.7	
Sitting with someone	53	77.9	49	74.2	
In front of a TV/computer	2	2.9	6	9.1	
**Way of cooking**					0.58
Boiled	20	29.4	20	30.3	
Fried	39	57.4	35	53.0	
Grilled/oven	5	7.4	11	16.7	
Mixed methods	1	1.5			
**Use of extra salt**					0.62
Yes	16	23.5	18	27.3	
No	52	76.5	48	72.7	
**Use of diet products**					0.43
Yes	15	22.1	11	16.7	
No	53	77.9	55	83.3	
**Physical activity level**					0.10
Extremely low (never/rarely)	5	7.4	13	19.7	
Low (<2 times per week)	24	35.3	21	31.8	
Moderate (2–3 times per week)	30	44.1	25	37.9	
High (3–5 times per week)	8	11.8	7	10.6	
Extremely high (everyday)	1	1.5	-	-	
**Free time workout**					0.49
Yes	17	25.0	20	30.3	
No	51	75.0	46	69.7	

BMI = Body Mass Index, WHR = Waist to Hip Ratio, SBP = Systolic Blood Pressure, DBP = Diastolic Blood Pressure.

**Table 2:** Energy and macronutrient intake.

Variable	Fasters (*n* = 68)	Non-Fasters (*n* = 66)	*p*-Value
	Mean ± SD	Mean ± SD	
Energy (kcal)	1453.73 ± 364.80	1580.86 ± 453.16	0.07
Protein (g)	48.89 ± 17.74	54.70 ± 19.45	0.07
Carbohydrate (g)	150.94 ± 50.78	148.60 ± 53.90	0.79
Dietary fiber (g)	20.74 ± 8.17	22.12 ± 10.35	0.39
Soluble fiber (g)	2.19 ± 1.42	1.9 ± 1.43	0.41
Sugar total (g)	48.54 ± 30.20	46.87 ± 27.61	0.73
Monosaccharides (g)	16.48 ± 12.65	12.34 ± 8.28	0.027
Disaccharides (g)	14.48 ± 10.94	14.15 ± 9.61	0.85
Other carbs (g)	66.39 ± 24.16	67.34 ± 26.57	0.82
Fat (g)	77.87 ± 25.76	90.55 ± 26.94	0.006
Saturated fat (g)	19.47 ± 8.49	23.26 ± 9.24	0.015
Monounsaturated fat (g)	40.99 ± 16.10	47.18 ± 13.72	0.018
Polyunsaturated fat (g)	8.56 ± 3.32	10.36 ± 5.51	0.023
Trans fatty acids (g)	0.57 ± 0.45	2.38 ± 1.20	0.035
Cholesterol (mg)	131.77 ± 97.79	176.55 ± 104.28	0.011
ω-3 fatty acids (mg)	0.73 ± 0.60	0.74 ± 0.44	0.91
ω-6 fatty acids (mg)	4.83 ± 2.48	6.61 ± 4.36	0.004
Water (ltr)	708.37 ± 237.84	631.95 ± 228.81	0.06
Alcohol (g)	0.39 ± 2.32	1.07 ± 2.54	0.11
Caffeine (mg)	0.45 ± 2.26	1.75 ± 5.45	0.07

**Table 3:** Biochemical analysis.

Variable	Fasters (*n* = 68)	Non-Fasters (*n* = 66)	*p*-Value
	Mean ± SD	Mean ± SD	
Total cholesterol (mg/dL)	214 ± 50	229 ± 44	0.054
Triglycerides (mg/dL)	138 ± 55	170 ± 77	0.057
HDL cholesterol (mg/dL)	54 ± 19	59 ± 17	0.08
LDL cholesterol (mg/dL)	137 ± 71	119 ± 45	0.08
Glucose (mg/dL)	86 ± 17	95 ± 25	0.022

HDL = High-Density Lipoprotein, LDL = Low-Density Lipoprotein.

**Table 4:** Metabolic syndrome components.

Variable	Fasters (*n* = 68)	Non-Fasters (*n* = 66)	*p*-Value
	*n* (%)	*n* (%)	
WC > 88 cm	49 (72.1)	41 (62)	0.55
FBG ≥ 100 mg/dL	8 (11.8)	16 (24.2)	0.039
HDL cholesterol < 50 mg/dL	32 (47.1)	21 (31.8)	0.61
TG ≥ 150 mg/dL	22 (33.8)	31 (47)	0.58
BP ≥ 130/85 mmHg	9 (13.2)	24 (36.4)	0.041
MetS prevalence	16 (23.5)	20 (30.3)	0.052

WC: Waist Circumference, FBG: Fasting Blood Glucose, HDL: High Density Lipoprotein, TG: Triglycerides, BP: Blood Pressure, MetS: Metabolic Syndrome.

## Data Availability

The raw data supporting the conclusions of this study are available from the corresponding author upon request.
